# Supplementary figures and images for: Prediction of dengue annual incidence using seasonal climate variability in Bangladesh between 2000 and 2018
Source: PLOS Glob Public Health. 2022 May 9;2(5):e0000047. doi: 10.1371/journal.pgph.0000047 (PMC10021868; doi:10.1371/journal.pgph.0000047)

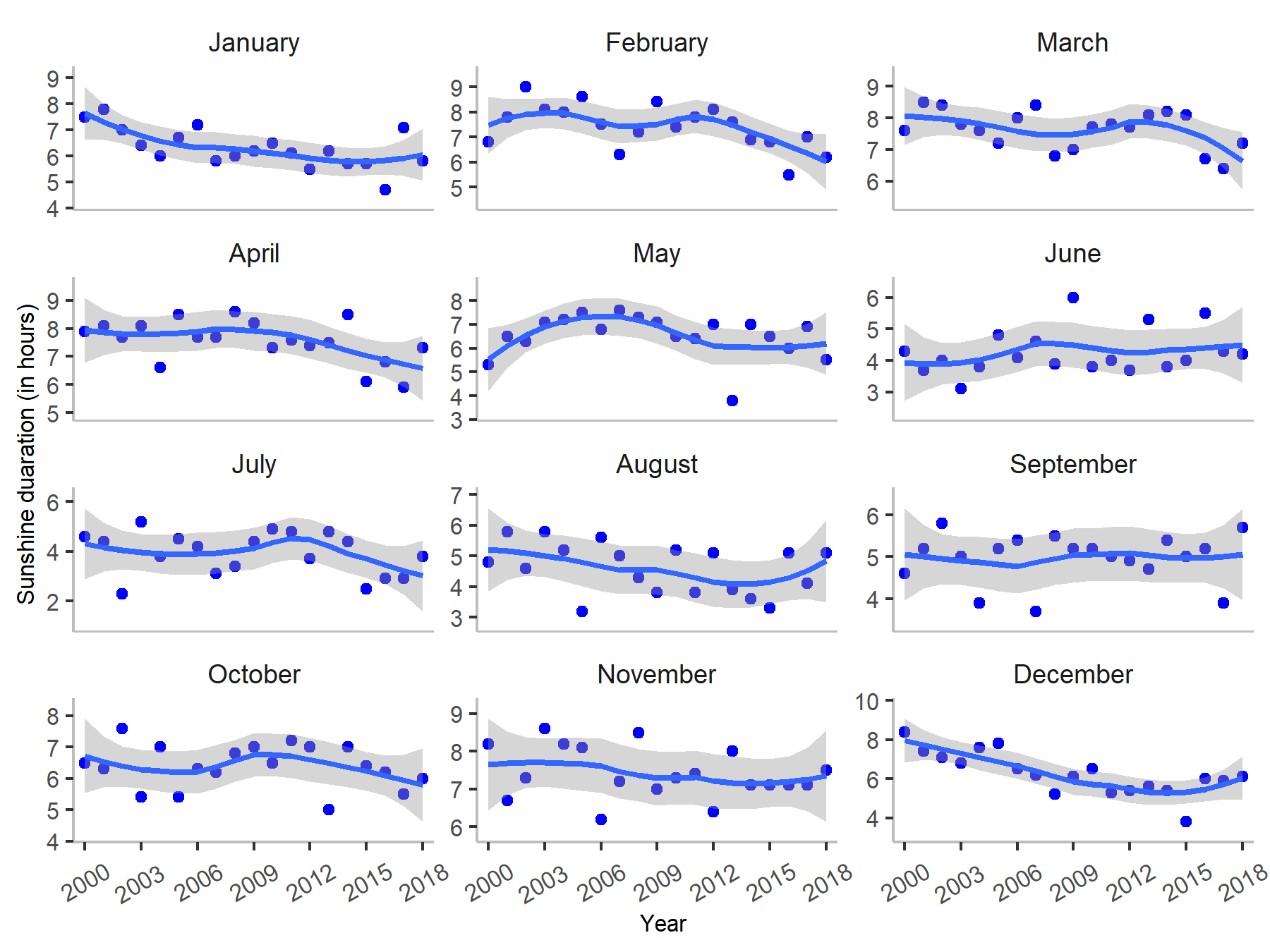

Supplement: S1 Fig — A LOESS smoothing function is used to obtain a smooth line to represent the trend over the years. The shaded region represents the 95% confidence interval. Dots represent the average sunshine duration of a given month for a particular year. (TIFF) [file pgph.0000047.s001.tiff]

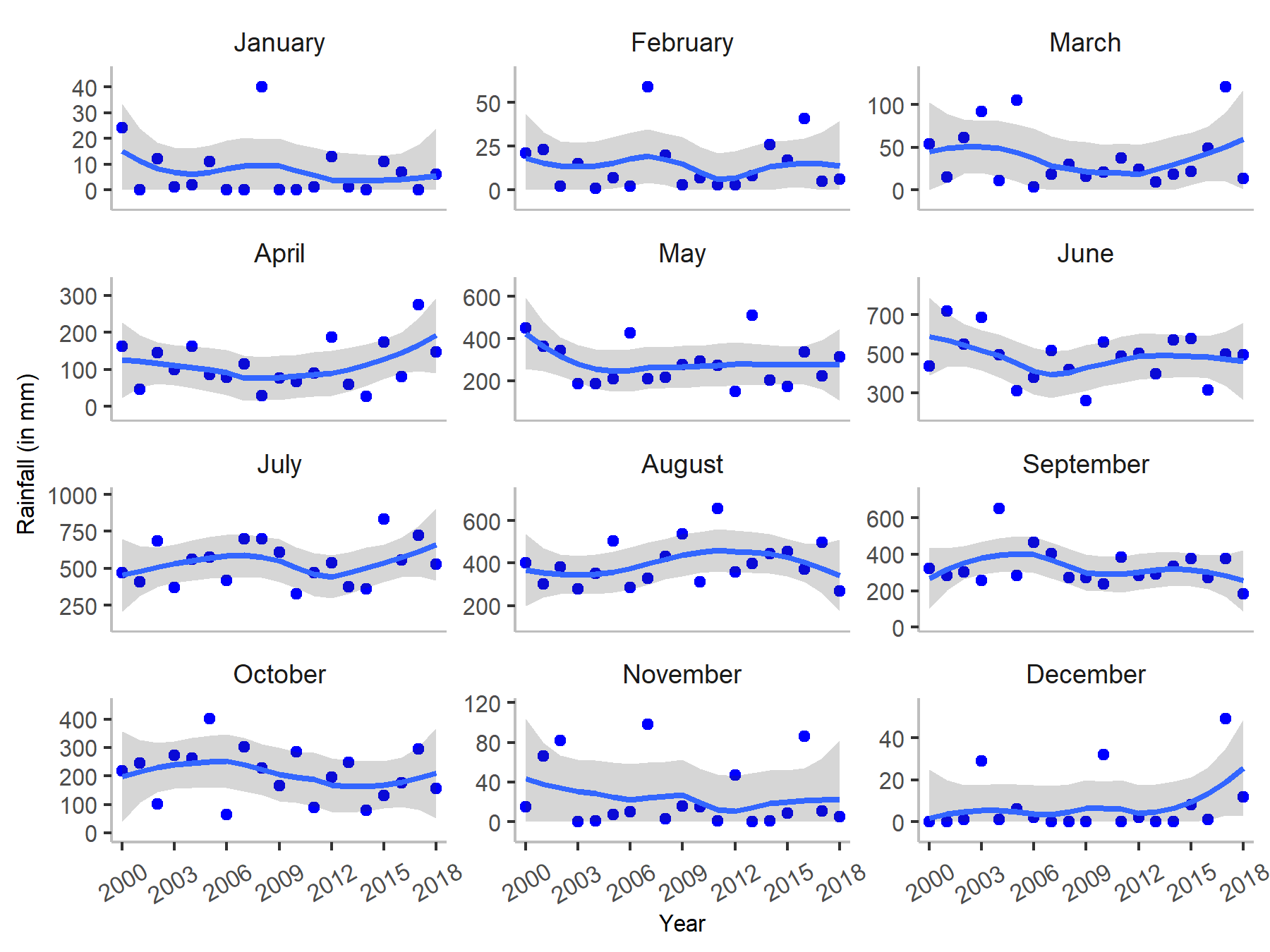

Supplement: S2 Fig — A LOESS smoothing function is used to obtain a smooth line to represent trend over years. The shaded region shows the 95% confidence interval. Dots represent the average rainfall of a given month for a particular year. (TIFF) [file pgph.0000047.s002.tiff]

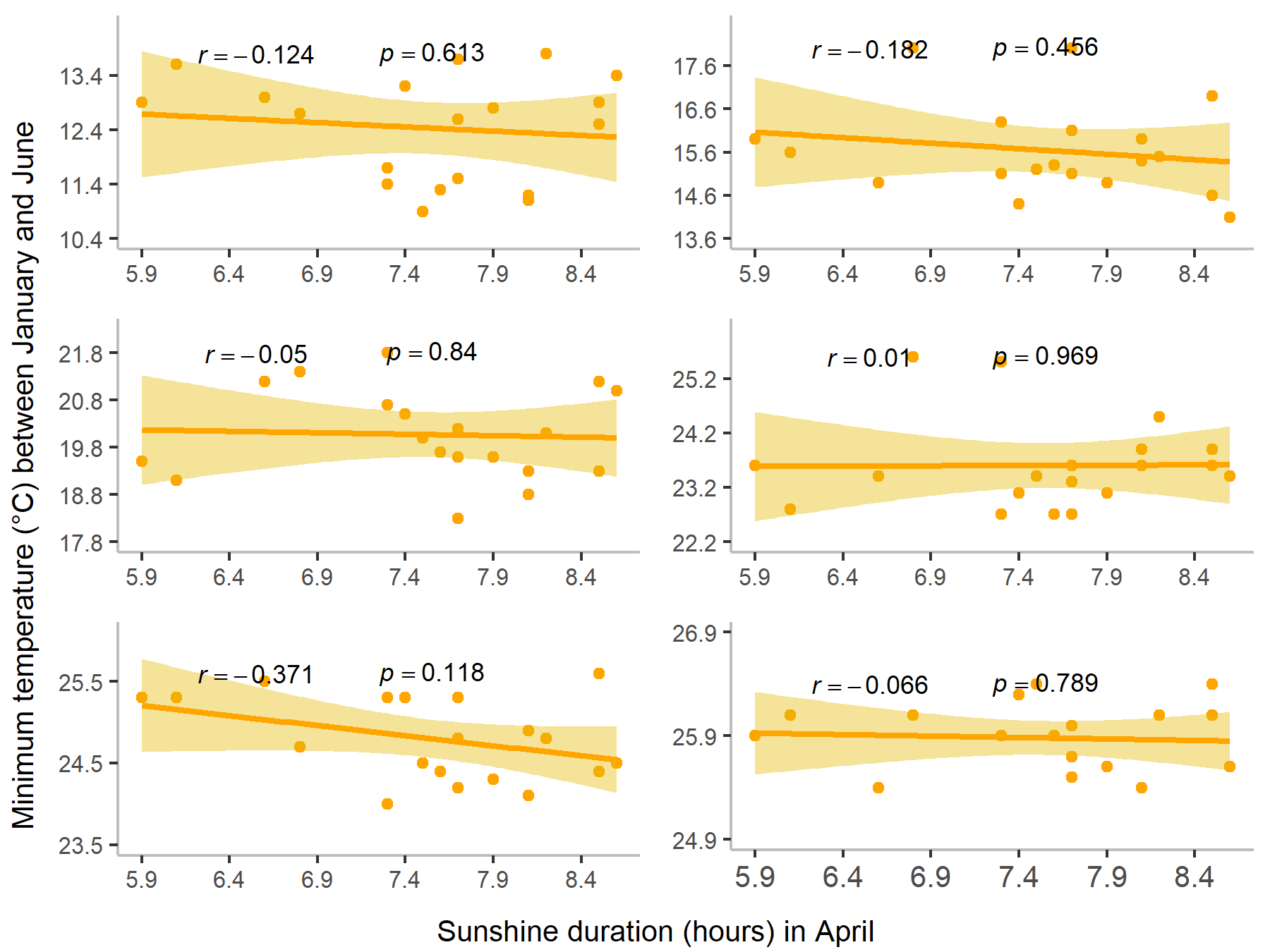

Supplement: S3 Fig — The top left panel represents minimum temperature data in January, whereas the bottom right represents minimum temperature data in June. r denotes the correlation coefficients score and p is p−value from a correlation test. The line refers to the regression line, and the shaded region shows the 95% confidence interval. The points are intersecting values of minimum temperature and sunshine duration. (TIFF) [file pgph.0000047.s003.tiff]

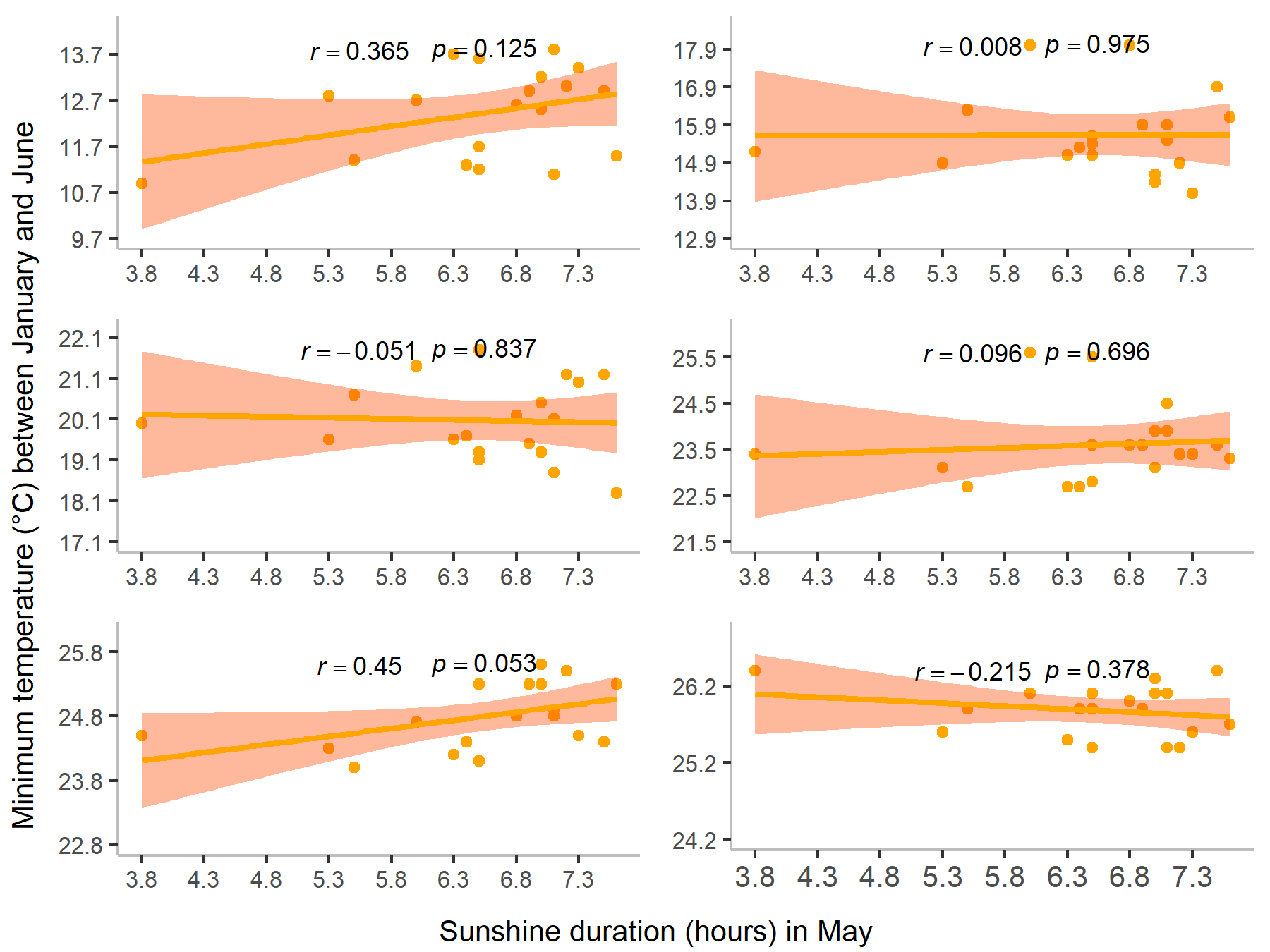

Supplement: S4 Fig — The top left panel represents minimum temperature data in January, whereas the bottom right represents minimum temperature data in June. r denotes the correlation coefficients score and p is the p−value from a correlation test. The line refers to the regression line, and the shaded region shows the 95% confidence interval. The points are intersecting values of minimum temperature and sunshine duration. (TIFF) [file pgph.0000047.s004.tiff]
