# Supplementary material for: Prediction of dengue annual incidence using seasonal climate variability in Bangladesh between 2000 and 2018
Source: PLOS Glob Public Health. 2022 May 9;2(5):e0000047. doi: 10.1371/journal.pgph.0000047 (PMC10021868; doi:10.1371/journal.pgph.0000047)
Supplement: S1 Table — (PDF) [file pgph.0000047.s005.pdf]

Table S1. Variance inflation factor (VIF) for the predictors used in the best prediction model: monthly minimum temperature, monthly sunshine duration, and monthly total rainfall.

Months	Minimum temperature	Sunshine duration	Total rainfall
January	3.42	-	3.85
February	2.88	-	2.80
March	3.96	-	-
April	3.06	5.64	3.98
May	4.07	3.28	-
June	2.13	-	3.29
Overall	2.86	3.46	6.48
