# Supplementary material for: Prediction of dengue annual incidence using seasonal climate variability in Bangladesh between 2000 and 2018
Source: PLOS Glob Public Health. 2022 May 9;2(5):e0000047. doi: 10.1371/journal.pgph.0000047 (PMC10021868; doi:10.1371/journal.pgph.0000047)
Supplement: S2 Table — ave.Ti, Si and tot.Ri represent mean temperature, sunshine duration and total rainfall in the ith month. For each of the variables included in the model, the corresponding AICc, the leave-one-out mean squared error for the validation set (MSEVa), the leave-one-out mean squared error for the training set (MSETr), and the mean squared error ratio (F=MSEvaMSETr) were calculated. (PDF) [file pgph.0000047.s006.pdf]

Table S2. (**Model 1**) Step-by-step forward selection results of the generalized Poisson regression model in each step based on AIC_c.$ave.T_i$, S_i and $tot.R_i$ represent mean temperature, sunshine duration and total rainfall in the ith month. For each of the variable included in the model, the corresponding AIC_c, the leave-one-out mean squared error for the validation set (MSE_{Va}), the leave-one-out mean squared error for the training set (MSE_{Tr}), and the mean squared error ratio $\left(F = \frac{MSE_{va}}{MSE_{Tr}}\right)$ were calculated.

Step	(Intercept)	$ave.T_5$	S_4	$tot.R_1$	f S_5	$ave.T_3$	$tot.R_4$	$tot.R_3$	$tot.R_6$	$ave.T_6$	$ave.T_1$	$ave.T_2$	$tot.R_5$	$tot.R_2$	S_6	AIC_c	MSE_{Va}	MSE_{Tr}	F
1	29.36	-0.75														29457	1.064	0.878	1.212
2	46.60	-1.11	-0.92													14522	0.518	0.386	1.341
3	48.40	-1.15	-1.06	0.0343												10507	0.395	0.266	1.483
4	65.26	-1.77	-1.36	0.0380	0.48											7151	0.446	0.260	1.715
5	59.14	-1.81	-1.41	0.0426	0.54	0.28										5684	0.528	0.303	1.741
6	48.52	-1.68	-1.13	0.0378	0.48	0.46	0.0041									4818	0.529	0.316	1.675
7	50.84	-1.71	-1.15	0.0391	0.53	0.41	0.0046	-0.0036								4445	0.575	0.306	1.882
8	61.03	-1.91	-1.25	0.0379	0.68	0.25	0.0048	-0.0067	-0.0017							3761	0.673	0.348	1.931
9	74.13	-1.83	-1.25	0.0300	0.57	0.33	0.0058	-0.0072	-0.0033	-0.55						2902	0.740	0.392	1.890
10	79.97	-1.84	-1.30	0.0333	0.62	0.30	0.0057	-0.0062	-0.0037	-0.64	-0.12					2719	0.762	0.379	2.009
11	73.70	-1.56	-1.11	0.0432	0.45	0.28	0.0069	-0.0060	-0.0032	-0.82	-0.18	0.22				2467	0.862	0.353	2.441
12	85.09	-1.30	-0.63	0.0329	-0.28	0.22	0.0082	-0.0072	-0.0051	-1.60	-0.05	0.50	-0.005			2005	0.772	0.301	2.562
13	81.87	-1.20	-0.35	0.0224	-0.46	0.29	0.0108	-0.0082	-0.0053	-1.75	0.02	0.54	-0.007	0.01		1751	1.039	0.245	4.239
14	86.27	-1.36	-0.37	0.0039	-0.61	0.30	0.0097	-0.0075	-0.0071	-1.64	0.18	0.46	-0.008	0.02	-0.46	1731	1.334	0.166	8.016
