# Supplementary material for: Prediction of dengue annual incidence using seasonal climate variability in Bangladesh between 2000 and 2018
Source: PLOS Glob Public Health. 2022 May 9;2(5):e0000047. doi: 10.1371/journal.pgph.0000047 (PMC10021868; doi:10.1371/journal.pgph.0000047)
Supplement: S3 Table — max.Ti, Si and tot.Ri represent maximum temperature, sunshine duration and total rainfall in the ith month. For each of the variables included in the model, the corresponding AICc, the leave-one-out mean squared error for the validation set (MSEVa), the leave-one-out mean squared error for the training set (MSETr), and the mean squared error ratio (F=MSEvaMSETr) were calculated. (PDF) [file pgph.0000047.s007.pdf]

Table S3. (**Model 2**) Step-by-step forward selection results of the generalized Poisson regression model in each step based on AIC_c. $max.T_i$, S_i and $tot.R_i$ represent maximum temperature, sunshine duration and total rainfall in the ith month. For each of the variable included in the model, the corresponding AIC_c, the leave-one-out mean squared error for the validation set (MSE_{Va}), the leave-one-out mean squared error for the training set (MSE_{Tr}), and the mean squared error ratio $\left(F = \frac{MSE_{va}}{MSE_{Tr}}\right)$ were calculated.

Step	(Intercept)	$max.T_5$	S_4	$tot.R_1$	S_5	$max.T_1$	$tot.R_6$	$tot.R_5$	S_6	$max.T_6$	$max.T_2$	$tot.R_4$	$tot.R_3$	$max.T_4$	AIC_c	MSE_{Va}	MSE_{Tr}	F
1	24.56	-0.50													32362	1.153	0.944	1.221
2	35.69	-0.67	-0.72												21627	0.807	0.549	1.471
3	37.57	-0.71	-0.86	0.03											17120	0.625	0.383	1.632
4	54.34	-1.25	-1.12	0.04	0.48										14477	0.753	0.356	2.115
5	77.66	-1.64	-1.44	0.05	0.81	-0.40									10918	0.840	0.458	1.833
6	102.47	-2.22	-1.85	0.06	1.33	-0.56	-0.0037								6959	1.150	0.664	1.731
7	105.22	-2.22	-2.11	0.07	1.70	-0.75	-0.0032	0.004							5901	1.342	0.797	1.683
8	107.87	-2.32	-2.25	0.08	2.02	-0.91	-0.0011	0.006	0.48						5326	1.562	0.912	1.713
9	87.17	-2.21	-2.30	0.09	2.34	-1.03	0.0020	0.010	0.59	0.47					4990	1.576	0.815	1.934
10	83.18	-2.11	-2.24	0.10	2.25	-1.04	0.0022	0.010	0.60	0.40	0.110				4822	1.638	0.817	2.004
11	59.01	-1.50	-1.66	0.12	2.17	-1.38	0.0055	0.013	1.32	0.18	0.410	0.008			3833	1.569	0.875	1.793
12	61.40	-1.49	-1.52	0.12	2.07	-1.30	0.0048	0.012	1.44	0.01	0.441	0.010	-0.004		3603	1.731	0.859	2.015
13	104.94	-2.25	-1.62	0.09	1.73	-0.98	-0.0030	0.002	0.94	-0.94	0.414	0.015	-0.009	0.51	3235	1.873	0.886	2.114
