# Supplementary material for: Prediction of dengue annual incidence using seasonal climate variability in Bangladesh between 2000 and 2018
Source: PLOS Glob Public Health. 2022 May 9;2(5):e0000047. doi: 10.1371/journal.pgph.0000047 (PMC10021868; doi:10.1371/journal.pgph.0000047)
Supplement: S4 Table — Ti, Si and Ri represent minimum temperature, sunshine duration and total rainfall in the ith month. For each of the variables included in the model, the corresponding AICc, the leave-one-out mean squared error for the validation set (MSEVa), the leave-one-out mean squared error for the training set (MSETr), and the mean squared error ratio (F=MSEvaMSETr) were calculated. (PDF) [file pgph.0000047.s008.pdf]

Table S4. (**Model 3**) Step-by-step forward selection results of the generalized Poisson regression model in each step based on AIC_c. T_i, S_i and R_i represent minimum temperature, sunshine duration and total rainfall in the ith month. For each of the variable included in the model, the corresponding AIC_c, the leave-one-out mean squared error for the validation set (MSE_{Va}), the leave-one-out mean squared error for the training set (MSE_{Tr}), and the mean squared error ratio $\left(F = \frac{MSE_{va}}{MSE_{Tr}}\right)$ were calculated.

Step	(Intercept)	T_5	S_4	T_1	T_3	T_6	S_5	T_2	T_4	R_2	R_1	R_4	R_6	AIC_c	MSE_{Va}	MSE_{Tr}	F
1	33.51	-1.038												30314	1.124	0.913	1.230
2	59.24	-1.766	-1.051											10893	0.399	0.301	1.326
3	62.36	-2.014	-1.079	0.257										8311	0.376	0.257	1.463
4	58.08	-2.002	-1.082	0.280	0.186									7219	0.426	0.285	1.496
5	76.29	-1.914	-1.142	0.229	0.274	-0.816								5055	0.429	0.262	1.637
6	74.52	-1.594	-1.044	0.282	0.296	-1.052	-0.298							3763	0.419	0.238	1.759
7	75.47	-1.393	-0.930	0.289	0.233	-1.340	-0.375	0.151						3152	0.429	0.193	2.219
8	69.44	-0.894	-0.730	0.322	0.348	-1.537	-0.506	0.353	-0.329					1517	0.258	0.114	2.260
9	69.27	-0.822	-0.715	0.321	0.422	-1.511	-0.484	0.350	-0.507	0.013				930	0.162	0.078	2.067
10	68.84	-0.837	-0.683	0.368	0.456	-1.481	-0.498	0.325	-0.548	0.016	-0.007			901	0.211	0.066	3.181
11	65.63	-0.824	-0.584	0.374	0.482	-1.431	-0.501	0.317	-0.539	0.018	-0.011	0.001		840	0.268	0.062	4.308
12	57.58	-0.721	-0.478	0.408	0.539	-1.343	-0.555	0.357	-0.536	0.019	-0.010	0.002	0.001	791	0.294	0.051	5.726
