# Supplementary material for: Prediction of dengue annual incidence using seasonal climate variability in Bangladesh between 2000 and 2018
Source: PLOS Glob Public Health. 2022 May 9;2(5):e0000047. doi: 10.1371/journal.pgph.0000047 (PMC10021868; doi:10.1371/journal.pgph.0000047)
Supplement: S5 Table — ave.Ti, Si and max.Ri represent mean temperature, sunshine duration and maximum rainfall in the ith month. For each of the variables included in the model, the corresponding AICc, the leave-one-out mean squared error for the validation set (MSEVa), the leave-one-out mean squared error for the training set (MSETr), and the mean squared error ratio (F=MSEvaMSETr) were calculated. (PDF) [file pgph.0000047.s009.pdf]

Table S5. (**Model 4**) Step-by-step forward selection results of the generalized Poisson regression model in each step based on AIC_c. $ave.T_i$, S_i and $max.R_i$ represent mean temperature, sunshine duration and maximum rainfall in the ith month. For each of the variable included in the model, the corresponding AIC_c, the leave-one-out mean squared error for the validation set (MSE_{Va}), the leave-one-out mean squared error for the training set (MSE_{Tr}), and the mean squared error ratio $\left(F = \frac{MSE_{va}}{MSE_{Tr}}\right)$ were calculated.

Step	(Intercept)	$ave.T_5$	S_4	$max.R_1$	S_5	$ave.T_3$	$max.R_4$	$ave.T_6$	$max.R_6$	$max.R_3$	$max.R_2$	$ave.T_4$	$ave.T_2$	$max.R_5$	AIC_c	MSE_{Va}	MSE_{Tr}	F
1	29.36	-0.75													29457	1.064	0.878	1.212
2	46.60	-1.11	-0.92												14522	0.518	0.386	1.341
3	47.90	-1.14	-1.04	0.055											10483	0.397	0.265	1.499
4	64.93	-1.76	-1.34	0.061	0.48										7136	0.453	0.264	1.717
5	58.93	-1.79	-1.38	0.066	0.53	0.26									5890	0.525	0.298	1.764
6	49.97	-1.69	-1.15	0.067	0.46	0.42	0.017								5358	0.591	0.336	1.761
7	49.46	-1.49	-1.01	0.068	0.29	0.63	0.028	-0.38							4660	0.709	0.375	1.893
8	56.57	-1.55	-1.07	0.062	0.30	0.63	0.029	-0.53	-0.0072						4194	0.818	0.432	1.892
9	63.33	-1.70	-1.15	0.071	0.42	0.53	0.034	-0.52	-0.0111	-0.0153					3279	0.918	0.476	1.927
10	57.91	-1.63	-0.88	0.068	0.38	0.64	0.052	-0.58	-0.0104	-0.0214	0.0183				2851	1.023	0.442	2.312
11	56.22	-1.43	-0.71	0.051	0.30	0.78	0.050	-0.58	-0.0117	-0.0154	0.0360	-0.31			2595	1.000	0.407	2.460
12	46.07	-0.95	-0.34	0.064	-0.02	0.84	0.057	-0.90	-0.0090	-0.0154	0.0426	-0.44	0.30		2169	0.999	0.386	2.586
13	45.16	-0.65	-0.02	0.072	-0.39	0.80	0.072	-1.39	-0.0122	-0.0181	0.0432	-0.31	0.51	-0.01	2026	1.022	0.394	2.592
