# Supplementary material for: Prediction of dengue annual incidence using seasonal climate variability in Bangladesh between 2000 and 2018
Source: PLOS Glob Public Health. 2022 May 9;2(5):e0000047. doi: 10.1371/journal.pgph.0000047 (PMC10021868; doi:10.1371/journal.pgph.0000047)
Supplement: S6 Table — max.Ti, Si and max.Ri represent maximum temperature, sunshine duration and maximum rainfall in the ith month. For each of the variables included in the model, the corresponding AICc, the leave-one-out mean squared error for the validation set (MSEVa), the leave-one-out mean squared error for the training set (MSETr), and the mean squared error ratio (F=MSEvaMSETr) were calculated. (PDF) [file pgph.0000047.s010.pdf]

Table S6. (**Model 5**) Step-by-step forward selection results of the generalized Poisson regression model in each step based on AIC_c. $max.T_i$, S_i and $max.R_i$ represent maximum temperature, sunshine duration and maximum rainfall in the ith month. For each of the variable included in the model, the corresponding AIC_c, the leave-one-out mean squared error for the validation set (MSE_{Va}), the leave-one-out mean squared error for the training set (MSE_{Tr}), and the mean squared error ratio $\left(F = \frac{MSE_{va}}{MSE_{Tr}}\right)$ were calculated.

Step	(Intercept)	$max.T_5$	S_4	$max.R_1$	S_5	$max.T_1$	$max.R_6$	$max.R_5$	S_6	$max.R_3$	$max.T_2$	$max.R_4$	$max.T_6$	$max.T_3$	$max.R_2$	AIC_c	MSE_{Va}	MSE_{Tr}	F
1	24.56	-0.50														32362	1.153	0.944	1.221
2	35.69	-0.67	-0.72													21627	0.807	0.549	1.471
3	37.43	-0.71	-0.85	0.06												16823	0.622	0.375	1.656
4	55.06	-1.27	-1.13	0.06	0.50											14041	0.748	0.355	2.105
5	75.63	-1.62	-1.40	0.08	0.79	-0.36										10991	0.836	0.446	1.875
6	95.35	-2.07	-1.77	0.08	1.15	-0.46	-0.016									8549	1.231	0.664	1.853
7	106.00	-2.25	-2.02	0.10	1.48	-0.71	-0.013	0.016								7505	1.491	0.810	1.841
8	116.58	-2.56	-2.21	0.12	1.96	-0.96	-0.003	0.025	0.54							6037	1.723	0.953	1.807
9	116.96	-2.64	-2.25	0.13	2.04	-0.87	-0.004	0.023	0.59	-0.011						5578	1.777	0.957	1.856
10	109.73	-2.50	-2.17	0.15	1.95	-0.93	-0.001	0.027	0.54	-0.009	0.12					5380	1.846	0.913	2.022
11	97.07	-2.25	-1.84	0.17	1.87	-1.07	0.006	0.035	0.67	-0.013	0.24	0.02				4872	2.162	0.879	2.459
12	99.56	-1.99	-1.51	0.19	1.59	-1.15	0.005	0.031	1.04	-0.020	0.45	0.04	-0.56			4432	2.896	1.090	2.657
13	89.20	-1.89	-1.39	0.18	1.52	-1.12	0.003	0.031	0.88	-0.013	0.33	0.05	-0.49	0.23		4291	3.217	0.956	3.367
14	75.70	-1.71	-0.92	0.16	1.15	-0.93	-0.009	0.019	0.40	-0.016	0.34	0.08	-0.53	0.42	0.03	3913	9.728	0.683	14.240
