# Supplementary material for: Prediction of dengue annual incidence using seasonal climate variability in Bangladesh between 2000 and 2018
Source: PLOS Glob Public Health. 2022 May 9;2(5):e0000047. doi: 10.1371/journal.pgph.0000047 (PMC10021868; doi:10.1371/journal.pgph.0000047)
Supplement: S7 Table — min.Ti, Si and max.Ri represent minimum temperature, sunshine duration and maximum rainfall in the ith month. For each of the variables included in the model, the corresponding AICc, the leave-one-out mean squared error for the validation set (MSEVa), the leave-one-out mean squared error for the training set (MSETr), and the mean squared error ratio (F=MSEvaMSETr) were calculated. (PDF) [file pgph.0000047.s011.pdf]

Table S7. (**Model 6**) Step-by-step forward selection results of the generalized Poisson regression model in each step based on AIC_c. $min.T_i$, S_i and $max.R_i$ represent minimum temperature, sunshine duration and maximum rainfall in the ith month. For each of the variable included in the model, the corresponding AIC_c, the leave-one-out mean squared error for the validation set (MSE_{Va}), the leave-one-out mean squared error for the training set (MSE_{Tr}), and the mean squared error ratio $\left(F = \frac{MSE_{va}}{MSE_{Tr}}\right)$ were calculated.

Step	(Intercept)	$min.T_5$	S_4	$min.T_1$	$min.T_3$	$min.T_6$	S_5	$min.T_2$	$min.T_4$	$max.R_2$	$max.R_4$	$max.R_6$	$max.R_1$	S_6	AIC_c	MSE_{Va}	MSE_{Tr}	F
1	33.51	-1.04													30314	1.12	0.91	1.23
2	59.24	-1.77	-1.05												10893	0.40	0.30	1.33
3	62.36	-2.01	-1.08	0.26											8311	0.38	0.26	1.46
4	58.08	-2.00	-1.08	0.28	0.19										7219	0.43	0.28	1.50
5	76.29	-1.91	-1.14	0.23	0.27	-0.82									5055	0.43	0.26	1.64
6	74.52	-1.59	-1.04	0.28	0.30	-1.05	-0.30								3763	0.42	0.24	1.76
7	75.47	-1.39	-0.93	0.29	0.23	-1.34	-0.38	0.15							3152	0.43	0.19	2.22
8	69.44	-0.89	-0.73	0.32	0.35	-1.54	-0.51	0.35	-0.33						1517	0.26	0.11	2.26
9	69.21	-0.75	-0.68	0.32	0.44	-1.56	-0.49	0.37	-0.56	0.02					899	0.15	0.07	2.15
10	66.33	-0.70	-0.56	0.31	0.47	-1.55	-0.51	0.38	-0.57	0.03	0.007				813	0.14	0.07	2.18
11	60.01	-0.57	-0.44	0.33	0.52	-1.55	-0.56	0.42	-0.57	0.03	0.010	0.0048			709	0.14	0.05	2.73
12	60.54	-0.60	-0.41	0.37	0.55	-1.51	-0.56	0.39	-0.64	0.04	0.011	0.0036	-0.01		693	0.20	0.04	4.75
13	57.67	-0.68	-0.36	0.44	0.61	-1.31	-0.61	0.35	-0.67	0.05	0.014	0.0004	-0.03	-0.23	675	0.31	0.03	9.82
