# Supplementary material for: Prediction of dengue annual incidence using seasonal climate variability in Bangladesh between 2000 and 2018
Source: PLOS Glob Public Health. 2022 May 9;2(5):e0000047. doi: 10.1371/journal.pgph.0000047 (PMC10021868; doi:10.1371/journal.pgph.0000047)
Supplement: S8 Table — Achieved by omitting the jth year in the jth iteration, where j = 1, …, 19, and j = 1 indicate the year 2000, j = 2 indicates 2001, etc. Italic font denotes the predicted annual dengue cases when the jth year is removed. (PDF) [file pgph.0000047.s012.pdf]

Table S8. Leave-one-out cross-validation (loocv) results for **Model 6** by omitting the jth year in the jth iteration, where $j = 1, \ldots, 19$, and $j = 1$ indicates the year 2000, $j = 2$ indicates 2001 and so on. Bold-italic font represents the predicted annual dengue cases when the jth year is removed.

		Year omitted in the jth iteration																		
Year	Observed cases	2000	2001	2002	2003	2004	2005	2006	2007	2008	2009	2010	2011	2012	2013	2014	2015	2016	2017	2018
2000	5551	***4122***	5491	5632	5429	5445	5414	5396	5473	5403	5489	5305	5356	5436	5447	5373	5503	5540	5343	5408
2001	2430	2213	***1642***	2419	2186	2281	2172	2160	2400	2140	2155	2231	2358	2126	2119	2063	2196	2271	2155	2084
2002	6232	6496	6411	***9933***	6609	6533	6641	6631	6330	6639	6496	6590	6498	6656	6602	6627	6537	6494	6669	6618
2003	487	490	452	459	***590***	583	550	528	606	549	556	614	459	529	540	551	560	406	511	556
2004	3934	3834	3905	3900	3796	***2217***	3849	3800	3996	3812	3807	3869	3829	3830	3794	3768	3875	3809	3779	3769
2005	1047	1003	1039	973	967	1103	***872***	956	1097	977	902	963	931	1075	948	946	1045	1004	946	903
2006	2200	2189	2268	2254	2221	2129	2148	***2020***	2126	2171	2182	2176	2306	2125	2174	2144	2133	2255	2218	2176
2007	466	664	596	563	689	593	661	682	***1790***	678	651	599	609	660	683	632	639	671	682	681
2008	1153	1083	1157	1091	1169	1099	1131	1175	1120	***1204***	1124	1217	1275	1125	1136	1208	1073	1136	1225	1169
2009	474	426	382	512	368	369	347	371	441	374	***287***	375	364	340	409	396	409	388	342	419
2010	409	595	475	493	569	480	548	546	361	550	540	***713***	434	585	574	532	554	604	532	555
2011	1359	1520	1407	1427	1488	1492	1515	1494	1408	1512	1510	1439	***3270***	1523	1524	1474	1534	1469	1464	1512
2012	671	771	809	817	792	766	756	809	738	796	837	847	832	***1054***	796	682	769	739	788	806
2013	1773	1745	1871	1719	1823	1869	1850	1832	1846	1825	1727	1905	1906	1821	***2150***	1781	1784	1778	1865	1769
2014	351	196	173	210	201	180	198	198	266	199	214	207	229	257	211	***151***	188	221	230	237
2015	3195	3176	3144	3231	3072	3193	3120	3067	3232	3090	3138	3065	3008	3118	3105	3036	***2272***	3118	3011	3068
2016	6213	6257	6246	6223	6278	6312	6305	6304	6297	6312	6302	6354	6268	6279	6300	6289	6297	***14014***	6299	6313
2017	2635	2464	2562	2472	2565	2460	2514	2549	2510	2521	2462	2563	2704	2559	2501	2663	2428	2581	***2121***	2535
2018	10169	10075	9933	10124	10041	9930	9983	10049	10037	10048	10183	10022	10024	10034	10112	10233	10030	10051	10054	***8440***
