# Supplementary material for: Prediction of dengue annual incidence using seasonal climate variability in Bangladesh between 2000 and 2018
Source: PLOS Glob Public Health. 2022 May 9;2(5):e0000047. doi: 10.1371/journal.pgph.0000047 (PMC10021868; doi:10.1371/journal.pgph.0000047)
Supplement: S9 Table — Achieved by omitting the jth year in the jth iteration, where j = 1, …, 19, and j = 1 indicates the year 2000, j = 2 indicates 2001, etc. Italic font denotes the predicted annual dengue cases when the jth year is removed. (PDF) [file pgph.0000047.s013.pdf]

Table S9. Leave-one-out cross-validation (loocv) results for **Model 3** by omitting the jth year in the jth iteration, where $j = 1, \ldots, 19$, and $j = 1$ indicates the year 2000, $j = 2$ indicates 2001 and so on. Bold-italic font represents the predicted annual dengue cases when the jth year is removed.

		Year omitted in the jth iteration																		
Year	Observed cases	2000	2001	2002	2003	2004	2005	2006	2007	2008	2009	2010	2011	2012	2013	2014	2015	2016	2017	2018
2000	5551	***4047***	5491	5604	5367	5449	5349	5320	5636	5354	5321	5245	5304	5321	5384	5305	5342	5307	5230	5321
2001	2430	2190	***1459***	2357	2152	2256	2107	2093	2336	2086	2083	2230	2089	2073	2073	2024	2085	2071	2127	2088
2002	6232	6492	6382	***9731***	6649	6490	6677	6689	6266	6673	6671	6589	6691	6693	6641	6671	6666	6710	6743	6697
2003	487	512	438	498	***634***	627	582	561	590	577	579	665	575	575	563	557	579	589	525	573
2004	3934	3812	3876	3877	3741	***1796***	3799	3753	3966	3770	3759	3888	3760	3760	3748	3720	3767	3761	3743	3764
2005	1047	1025	1038	1001	954	1149	***880***	954	1079	978	954	980	965	989	937	904	975	958	968	973
2006	2200	2209	2291	2235	2255	2101	2152	***2087***	2129	2179	2184	2137	2185	2170	2196	2166	2176	2178	2220	2185
2007	466	658	631	595	728	611	709	725	***2258***	712	722	608	726	726	708	649	717	718	740	721
2008	1153	1070	1156	1094	1181	1099	1155	1181	1031	***1290***	1178	1211	1188	1178	1145	1224	1164	1175	1253	1173
2009	474	476	466	603	440	453	430	452	481	452	***440***	447	458	455	480	440	468	444	408	442
2010	409	694	558	600	696	532	661	670	446	671	667	***932***	662	681	692	620	673	654	642	664
2011	1359	1487	1278	1327	1379	1355	1378	1383	1534	1416	1372	1308	***1408***	1369	1444	1441	1386	1405	1287	1393
2012	671	693	755	700	710	689	667	718	740	710	709	823	705	***743***	714	627	697	726	686	714
2013	1773	1734	1863	1720	1825	1882	1864	1838	1715	1823	1831	1934	1852	1843	***2169***	1764	1827	1841	1917	1845
2014	351	164	144	173	170	143	159	166	265	163	166	191	165	173	178	***132***	164	169	194	164
2015	3195	3278	3192	3346	3155	3259	3195	3162	3283	3188	3182	3097	3167	3185	3205	3116	***2964***	3157	3085	3164
2016	6213	6162	6128	6105	6152	6206	6181	6188	6283	6197	6185	6303	6183	6173	6192	6231	6183	***5721***	6168	6196
2017	2635	2420	2573	2431	2543	2467	2506	2509	2428	2477	2492	2555	2514	2510	2455	2610	2484	2497	***1905***	2504
2018	10169	10122	10060	10253	10164	10046	10131	10189	10075	10170	10219	10128	10201	10203	10220	10328	10200	10179	10180	***10445***
