# Supplementary material for: Prediction of dengue annual incidence using seasonal climate variability in Bangladesh between 2000 and 2018
Source: PLOS Glob Public Health. 2022 May 9;2(5):e0000047. doi: 10.1371/journal.pgph.0000047 (PMC10021868; doi:10.1371/journal.pgph.0000047)
Supplement: S10 Table — SD represents the standard deviations of the estimate of each predictor. Asterisks in the p−value indicates that the predictors are significant with certain levels (i.e. ** = 0.001; * = 0.01). (PDF) [file pgph.0000047.s014.pdf]

3 Parameters estimation and prediction based on quasi Poisson regression model

Table S10. Parameter estimates of the best prediction model based on quasi Poisson regression. SD represents the standard deviations of the estimate of each predictor. Asterisks in the $p-$value indicates that the predictors are significant with certain levels (i.e. ** = 0.001; * = 0.01).

Predictors	Estimates	SD	$p-$value
Intercept	57.5835	11.0392	0.0020**
T_1	0.4082	0.0974	0.0057**
T_2	0.3571	0.0948	0.0093**
T_3	0.5386	0.1110	0.0028**
T_4	-0.5362	0.1185	0.0040**
T_5	-0.7209	0.2284	0.0197*
T_6	-1.3425	0.2407	0.0014**
S_4	-0.4778	0.1954	0.0501
S_5	-0.5551	0.1128	0.0027**
R_1	-0.0104	0.0116	0.4044
R_2	0.0191	0.0073	0.0393*
R_4	0.0020	0.0017	0.2696
R_6	0.0009	0.0010	0.4061
