# Supplementary material for: Prediction of dengue annual incidence using seasonal climate variability in Bangladesh between 2000 and 2018
Source: PLOS Glob Public Health. 2022 May 9;2(5):e0000047. doi: 10.1371/journal.pgph.0000047 (PMC10021868; doi:10.1371/journal.pgph.0000047)
Supplement: S11 Table — The lower and upper boundaries represent the lower and upper limit of the 95% bootstrap confidence interval, respectively, for the predicted value. (PDF) [file pgph.0000047.s015.pdf]

Table S11. Comparison between observed and predicted annual dengue cases in Bangladesh between 2000 and 2018. The lower bound and the upper bound represents the lower and upper limit of 95% bootstrap confidence interval respectively for the predicted value.

Year	2000	2001	2002	2003	2004	2005	2006	2007	2008	2009	2010	2011	2012	2013	2014	2015	2016	2017	2018
Observed	5551	2430	6232	487	3934	1047	2200	466	1153	474	409	1359	671	1773	351	3195	6213	2635	10169
Predicted	5141	2607	8146	438	2473	1340	2100	1549	893	618	346	1474	743	1892	291	3416	5698	1807	10078
Lower bound	5024	2535	7963	413	2394	1293	2020	1466	840	587	323	1423	709	1814	276	3308	5554	1735	9899
Upper bound	5251	2692	8330	464	2550	1393	2185	1631	959	646	371	1525	780	1970	309	3516	5846	1876	10253
