# Supplementary material for: Prediction of dengue annual incidence using seasonal climate variability in Bangladesh between 2000 and 2018
Source: PLOS Glob Public Health. 2022 May 9;2(5):e0000047. doi: 10.1371/journal.pgph.0000047 (PMC10021868; doi:10.1371/journal.pgph.0000047)
Supplement: S12 Table — (PDF) [file pgph.0000047.s016.pdf]

**Table S12.** Comparison of negative binomial regression models based on $AIC_c$.

| Model No. | Temperature | Rainfall | Sunshine | Expression | $AIC_c$ |
|---|---|---|---|---|---|
| NB 1 | $ave.T_i$ | $tot.R_i$ | $S$ | $S_5 + R_4 + T_5 + S_4 + R_1$ | 332 |
| NB 2 | $max.T_i$ | $tot.R_i$ | $S$ | $S_5 + T_4$ | 334 |
| NB 3 | $min.T_i$ | $tot.R_i$ | $S$ | $S_5 + R_4 + T_5 + S_4$ | 331 |
| NB 4 | $ave.T_i$ | $max.R_i$ | $S$ | $S_5 + S_4 + T_5 + R_1$ | 327 |
| NB 5 | $max.T_i$ | $max.R_i$ | $S$ | $S_5 + T_4$ | 334 |
| NB 6 | $min.T_i$ | $max.R_i$ | $S$ | $S_5 + S_4 + T_5 + T_1$ | 327 |
