# Supplementary material for: Prediction of dengue annual incidence using seasonal climate variability in Bangladesh between 2000 and 2018
Source: PLOS Glob Public Health. 2022 May 9;2(5):e0000047. doi: 10.1371/journal.pgph.0000047 (PMC10021868; doi:10.1371/journal.pgph.0000047)
Supplement: S13 Table — (PDF) [file pgph.0000047.s017.pdf]

Table S13. Comparison of the validation results among the best fitting models in negative binomial regression.

Model	MSE_{Va}	MSE_{Tr}	F ratio
NB 4	0.54	0.235	2.28
NB 6	0.50	0.238	2.105
