# Supplementary material for: Prediction of dengue annual incidence using seasonal climate variability in Bangladesh between 2000 and 2018
Source: PLOS Glob Public Health. 2022 May 9;2(5):e0000047. doi: 10.1371/journal.pgph.0000047 (PMC10021868; doi:10.1371/journal.pgph.0000047)
Supplement: S1 Text — (DOCX) [file pgph.0000047.s019.docx]

**Prediction of dengue annual incidence using seasonal climate variability in Bangladesh between 2000 and 2018**

M. Pear Hossain^1,2^, Wen Zhou^3^, Chao Ren^4^, John Marshall^5^, Hsiang-Yu Yuan^1*^

1. Department of Biomedical Sciences, Jockey Club College of Veterinary Medicine and Life Sciences, City University of Hong Kong, Kowloon, Hong Kong
2. Department of Statistics, Bangabandhu Sheikh Mujibur Rahman Science and Technology University, Gopalganj, Bangladesh
3. School of Energy and Environment, City University of Hong Kong, Kowloon, Hong Kong
4. Faculty of Architecture, The University of Hong Kong, Pokfulam, Hong Kong
5. Division of Biostatistics, School of Public Health, University of California, Berkeley, USA

* corresponding author. Email: sean.yuan@cityu.edu.hk

# Weather trend over the years

Mosquitoes are usually active in darker environments. With an increase in sunshine duration, their activity decreases, and the reverse is true for shorter sunshine duration [1]. Therefore, sunshine duration is expected to has a significant impact on mosquito-borne viral diseases like dengue. Sunshine duration measured in hours was found to have a decreasing trend from January to April and in December for different years between 2000 and 2018. However, it was almost stable from June to November. In addition, sunshine duration was shorter during the monsoon compared to the winter months from December to March (S1 Fig). Therefore, mosquitoes were more active during the monsoon, causing faster dengue transmission, which is reflected by the number of dengue cases in Fig 3 in the main text.

Rainfall is a critical meteorological factor that causes standing water in small storage areas, which are considered potential breeding sites for *Aedes* mosquitoes [2]. Rainfall was higher from May to August compared to other months of the year (S2 Fig). On the other hand, from December to March, the total amount of rain was minimal. However, the chance of rain water persisting in potential mosquito habitat during this time was higher due to light rain.

Therefore, we found these three climate variables- minimum temperature, sunshine duration, and total rainfall- to be a good combination of climate variables capable of predicting dengue incidence optimally. We assumed that climate factors in the months prior to the dengue season had a significant impact on dengue transmission. Consequently, the minimum daily temperature and total daily rainfall from January to June and the daily sunshine duration from April to June were chosen as possible predictor variables for dengue prediction.

# Checking the association among the predictors

A previous study (not about dengue fever) conducted in Europe found that temperature and sunshine are correlated to some extent [3]. We have conducted a correlation test and a multicolliniearity test to check whether this possible correlation exists in our study area and can cause a multicollinearity issue in our model. We found that the minimum temperature and sunshine duration in different months had a very low correlation coefficient score with a maximum value of $r = 0.45$ in May. Moreover, none of the correlation scores was statistically significant (supplementary Figs S3 and S4). These indicate that there is no apparent correlation among these variables.

Furthermore, we checked the multicollinearity among the predictors using a statistical technique called variance inflation factor (VIF). As a rule of thumb, if VIF is less than 10, the multicollinearity does not exist, and the predictors can be kept in the model [4]. On the other hand, if the VIF score is high ($\geq$ 10), the predictor having the largest VIF should be removed from the model as a remedial measure. We applied two approaches to estimate the VIF. In the first approach we considered minimum temperature, sunshine duration and total rainfall for all months in different years together. We observed that the VIF score for minimum temperature was 2.86, and for sunshine duration, the score was 3.46 (supplementary S1 Table). In the second approach, we considered the monthly climate variables as predictors. In this case, we found that VIF score for the monthly climate predictors was less than 5 except sunshine duration in April (VIF = 5.64). Therefore, the inclusion of minimum temperature and sunshine do not create any multicollinearity issue in the prediction results.

# Modelling dengue based on the negative binomial regression

To obtain an appropriate set of predictors for predicting the number of annual dengue cases using negative binomial regression, we compared six models with different combinations of climate variables (S2 Table). The best prediction model was determined using a two-stage model selection approach. In the first stage, model NB 4 and model NB 6, were chosen based on the lowest values of $AIC_{c}$. In the second stage, *LOOCV* was conducted for the selected models. After $LOOCV$ was performed, model NB 6 was identified as the best prediction model among these six models, with the lowest mean squared error for the validation set, compared with model NB 4 (0.50 vs 0.54; see S3 Table). Note that the best prediction model (NB 6) based on negative binomial regression discarded the rainfall effect from the model. Finally, we compared the best prediction model NB 6 with the best prediction model (Model 3) obtained based on the Poisson regression model (in the main text, Table 2). Model 3 was chosen as the best prediction model among all models compared because Model 3 showed the best goodness-of-fit than others. Therefore, Model 3 was implemented for further analysis in the main text.

Models NB 1, 2 and 3 considered average, maximum and minimum monthly temperature, respectively, along with monthly sunshine duration and monthly total rainfall. Models NB 4, 5 and 6 considered average, maximum and minimum monthly temperature, respectively, with monthly sunshine duration and maximum monthly rainfall. Mean squared errors for the validation data set ($MSE_{Va}$) and the training data set ($MSE_{Tr}$) were used to select the best prediction model.

**References**

1. Kim YM, Park JW, Cheong HK. Estimated effect of climatic variables on the transmission of plasmodium vivax malaria in the republic of Korea. Environ Health Perspect. 2012;120: 1314–1319. doi:10.1289/ehp.1104577

2. Getachew D, Tekie H, Gebre-Michael T, Balkew M, Mesfin A. Breeding sites of aedes aegypti: Potential dengue vectors in dire Dawa, east Ethiopia. Interdiscip Perspect Infect Dis. 2015;2015. doi:10.1155/2015/706276

3. van den Besselaar EJM, Sanchez-Lorenzo A, Wild M, Klein Tank AMG, de Laat ATJ. Relationship between sunshine duration and temperature trends across Europe since the second half of the twentieth century. J Geophys Res Atmos. 2015;120: 10,823-10,836. doi:10.1002/2015JD023640

4. Kalnins A. Multicollinearity: How common factors cause Type 1 errors in multivariate regression. Strateg Manag J. 2018;39: 2362–2385. doi:10.1002/smj.2783
